# Diagnosis and Management of Synchronous Prostate and Rectal Cancer in a Patient

**DOI:** 10.7759/cureus.105182

**Published:** 2026-03-13

**Authors:** Hamail Iqbal, Nilanjan Haldar, Adam C Mueller

**Affiliations:** 1 Department of Medicine, Cooper Medical School of Rowan University, Camden, USA; 2 Department of Radiation Oncology, Thomas Jefferson University, Philadelphia, USA

**Keywords:** chemoradiation, field cancerization, prostate cancer, radiation therapy, rectal cancer

## Abstract

The management of the synchronous occurrence of prostate and rectal cancer presents a challenge due to their anatomic proximity. Considering the high morbidity and mortality of malignancies individually, well-targeted treatment planning is required for optimal survival outcomes; however, management strategies have not been standardized. We describe the diagnosis and treatment of synchronous prostate and rectal cancer in a single patient. An Epic chart review of a 70-year-old man with synchronous prostate and rectal cancer was completed. The patient presented with urinary frequency, weak urinary stream, and nocturia. Magnetic resonance imaging (MRI) and transrectal biopsy confirmed intermediate-risk, stage IIC prostate adenocarcinoma. Concurrently, findings suspicious for malignancy were identified on screening colonoscopy. MRI and pathology confirmed stage IIIB colonic adenocarcinoma. Initial treatment for the prostate cancer was begun with leuprolide and bicalutamide. Subsequently, the patient was prescribed volumetric modulated arc therapy (VMAT) to the rectum, prostate, and lymph nodes; followed by the rectum and prostate; and finally the prostate. The patient received concurrent capecitabine. When treating synchronous malignancies, an integrated approach is necessary to minimize the length of intervention and adverse events. Compared to the serial treatment of rectal and prostate cancer, concurrent treatment with the expansion of the target volume has demonstrated decreased risk of wound complications and comparable disease-free survival. Further long-term follow-up and monitoring are needed to establish standard management strategies for synchronous prostate and rectal cancer.

## Introduction

Prostate and rectal cancer are among the leading cancer diagnoses in men. Prostate cancer is the second leading cause of cancer-related death among men worldwide, with more than one million new cases diagnosed annually [1]. Rectal cancer, which accounts for roughly one-third of all colorectal cancers, also represents a major contributor to global cancer morbidity and mortality [1]. Due to the anatomic proximity of these two structures and their overall incidence in older patients, there is a moderate potential of individuals having both cancers within their lifetime [2-4]. However, the synchronous occurrence of prostate and rectal cancer, defined as both cancers diagnosed within a short interval, is exceedingly rare. A population-based registry from Sweden estimated an incidence of 0.1% among patients diagnosed with either rectal or prostate cancer between 1995 and 2011 [5]. More recent Swedish registry data indicate that among patients with prostate cancer from 1993 to 2019, 0.25% had synchronous rectal cancer, with age-standardized incidence rates rising over time [6]. Multi-institutional data from the United States also suggest an incidence of 0.17% using a broader definition of synchronous presentation, being diagnosis or treatment within 12 months [7]. Notably, the increasing use of pelvic magnetic resonance imaging (MRI) for rectal cancer staging has contributed to a rise in the incidental detection of synchronous prostate cancer, but the entity remains rare [5].

A synchronous presentation poses unique diagnostic and therapeutic challenges due to overlapping anatomy, the need for careful staging of two distinct malignancies, and the absence of standardized guidelines. The management of synchronous disease is particularly complex because optimal treatment strategies for each cancer can conflict with one another. Radical prostatectomy in the setting of locally advanced rectal cancer carries significant surgical morbidity, including risks of pelvic exenteration and functional impairment [8]. Likewise, the higher doses of radiation required for prostate cancer can increase rectal toxicity and complicate subsequent surgical intervention [9]. Thus, tailoring a strategy that balances oncologic control with the quality of life requires the careful consideration of available evidence. Here, we describe the workup and treatment of a patient with concurrent stage IIIB rectal adenocarcinoma and intermediate-risk, stage IIC prostate adenocarcinoma, emphasizing the clinical reasoning that informed the chosen management strategy.

## Case presentation

Diagnosis

A 70-year-old male patient presented to the urologist with complaints of urinary frequency, a weak urinary stream, and nocturia. He did not experience hematuria, fevers, chills, or dysuria. He had no significant medical history but was a current smoker and reported heavy alcohol use at the time of the visit.

**Figure 1:**
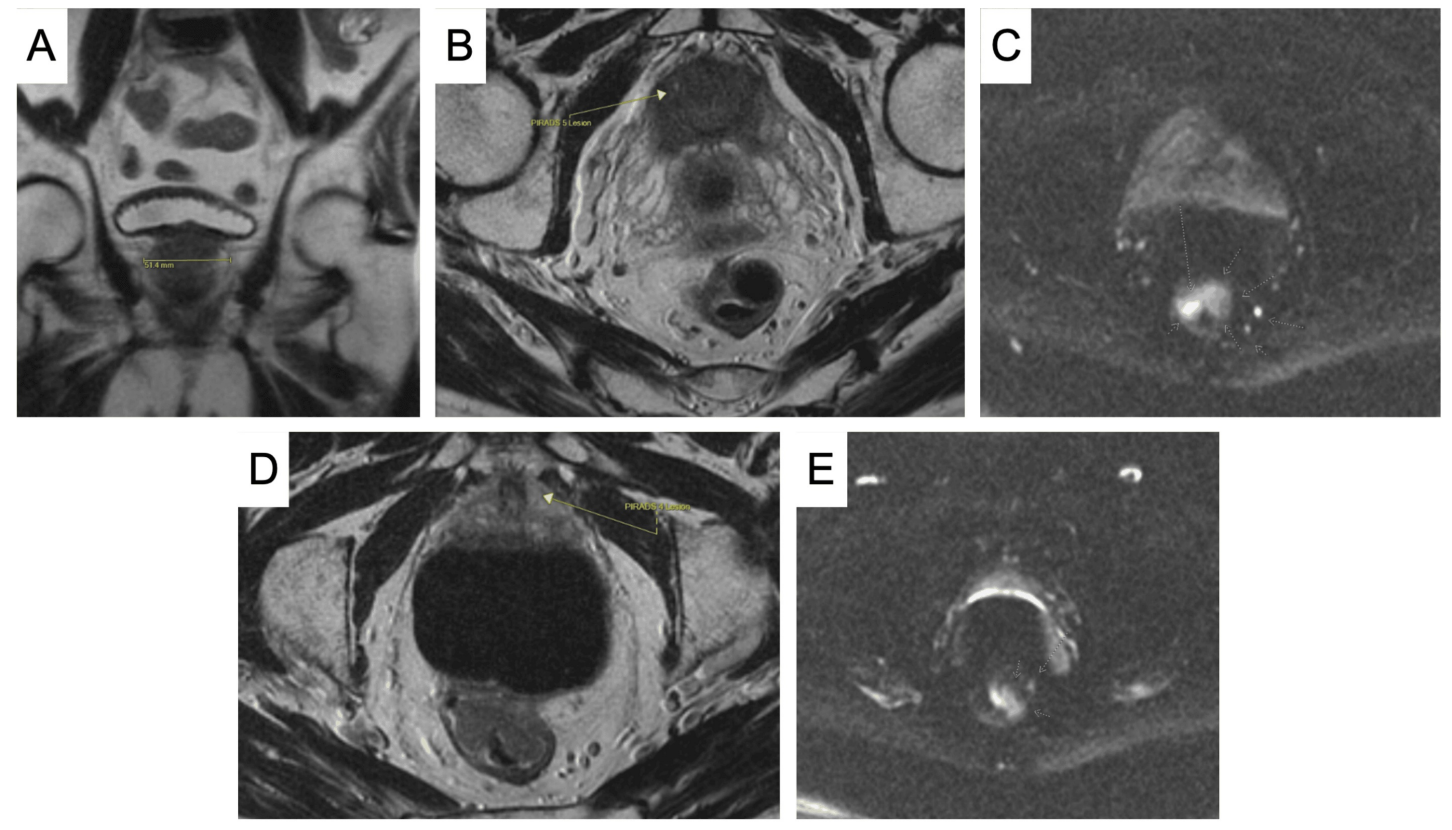
(A) Coronal MRI of the pelvis demonstrating a prostate measuring 5 cm transversely. (B) Axial MRI view and (C) diffusion-weighted image of a T2-weighted hypointense 1.7 cm nodule in the right mid-gland graded as PI-RADS 5. (D) Axial MRI view and (E) diffusion-weighted image of a T2-weighted hypointense 1 cm nodule arising from the midland to the apex of the left peripheral zone graded as PI-RADS 4. MRI, magnetic resonance imaging; PI-RADS, Prostate Imaging-Reporting and Data System

Laboratory examination revealed a prostate-specific antigen (PSA) of 8.0 ng/mL and a carcinoembryonic antigen (CEA) of 3 ng/mL. The patient subsequently underwent magnetic resonance imaging (MRI) of the pelvis with and without contrast. An enlarged prostate with an overall volume of 70 mL and measuring 5 cm transversely, with evidence of chronic bladder outlet obstruction, was appreciated (Figure 1A). Two T2-weighted hypointense nodules were identified: a 1.7 cm lesion in the right mid-gland graded as Prostate Imaging-Reporting and Data System (PI-RADS) 5 (Figure 1B, 1C) and a 1 cm enhancing lesion arising from the midland to the apex of the left peripheral zone graded as PI-RADS 4 (Figure 1D, 1E). The further evaluation of these lesions was done by transrectal MRI-fusion needle biopsy. Three targeted biopsy cores were taken from the visualized lesion of interest, followed by a 12-core systemic biopsy. Five out of 14 cores were positive and histologically demonstrated adenocarcinoma with a Gleason score of 7 (4+3) and grade group 3 with the loss of basal cell staining and strong alpha-methylacyl-CoA racemase (AMACR) staining. The patient was staged with a nuclear medicine bone scan that noted focal L5 uptake. A subsequent MRI of the lumbar spine found a right L5 superior endplate Schmorl’s node. Accordingly, the cancer was determined to be an unfavorable intermediate-risk prostate adenocarcinoma stage IIC, cT1, cN0, cMX.

**Figure 2:**
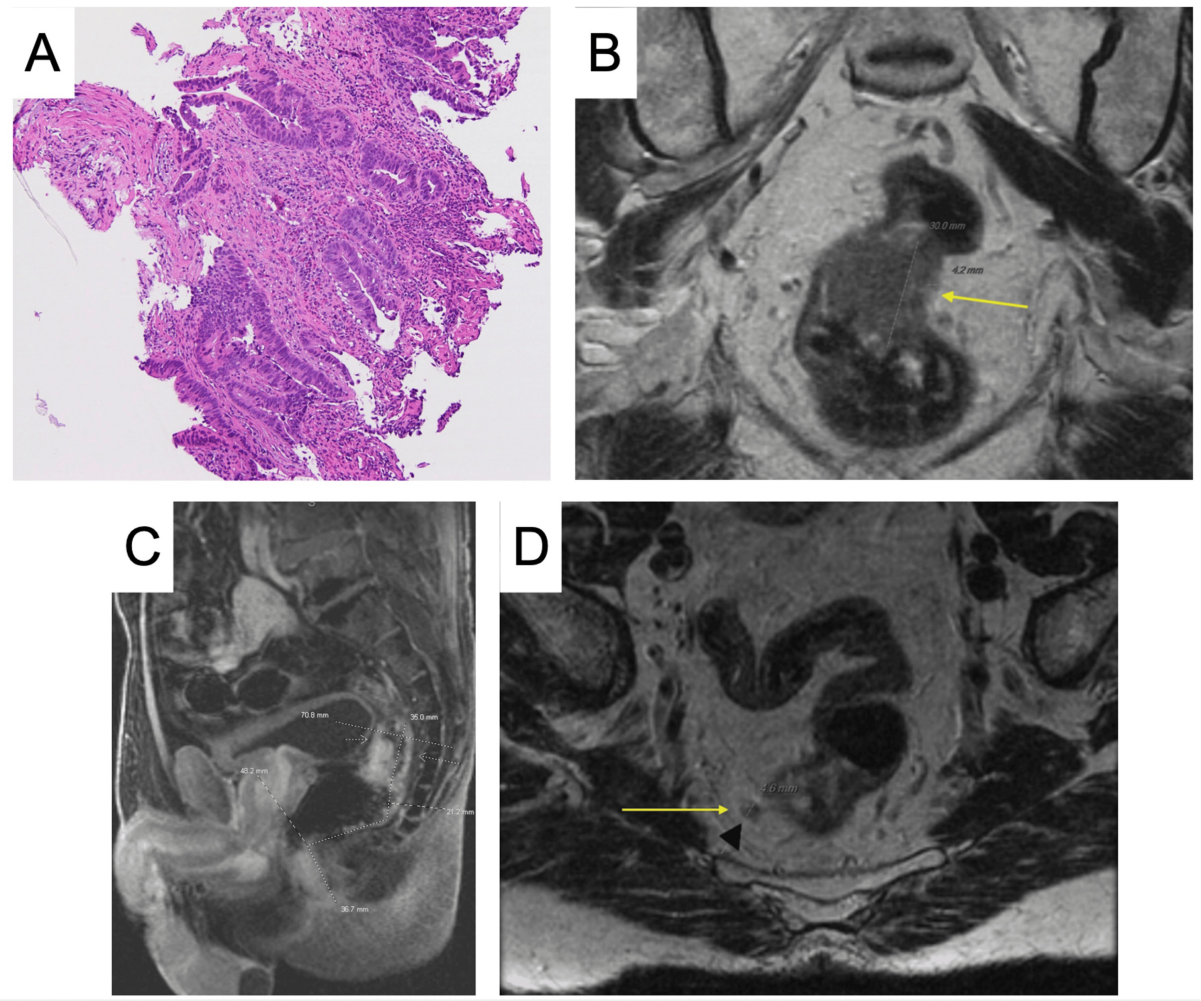
(A) Pathological evaluation of moderately differentiated invasive colonic adenocarcinoma. (B) MRI demonstrating a 3 cm non-mucinous semi-circumferential lesion involving the anterior rectal wall with a 4 mm extramural depth of invasion. (C) Sagittal view of an MRI of the abdomen and pelvis. (D) MRI demonstrating an involved mesorectal lymph node measuring 4.6 mm. MRI: magnetic resonance imaging

During the same time interval, findings suspicious for malignancy were identified on Cologuard screening (Exact Sciences Corp., Madison, WI) and colonoscopy. No gross hematochezia, melena, rectal pain, weight loss, or loss of appetite was reported by the patient at the time. He subsequently underwent an endoscopic submucosal dissection of a rectal polyp found on the colonoscopy. The pathological evaluation of the specimen revealed moderately differentiated invasive colonic adenocarcinoma (Figure 2A). Immunohistochemistry demonstrated an intact nuclear expression of mismatch repair proteins. Microsatellite instability testing was also negative.

Further radiological studies were performed, in which computed tomography (CT) imaging of the abdomen found no evidence of metastasis, while the CT of the chest found multiple pulmonary nodules with centrilobular emphysema. The latter findings were largely corroborated by the patient’s smoking history, alluding to no distant metastasis. An MRI of the pelvis with and without contrast was performed for the staging of the rectal cancer. A 3 cm non-mucinous semi-circumferential lesion was found involving the anterior rectal wall with a 4 mm extramural depth of invasion and a distance of 6.9 cm from the top of the sphincter complex (Figure 2B, 2C). Extramural venous invasion and mesorectal, superior rectal, internal iliac, and obturator lymph node involvement were also present, with a total of 11 involved lymph nodes (Figure 2D). Accordingly, the rectal cancer was determined to be stage IIIB, cT3, cN2, cM0. In relation to the prostate cancer, the rectal cancer was 36.7 mm apart.

Treatment

The patient initially began androgen deprivation therapy (ADT) for his prostate cancer with six months of leuprolide, along with tamsulosin for symptomatic relief. Radical robotic prostatectomy with bilateral pelvic lymph node dissection was considered. However, the procedure was ultimately not pursued due to the concurrent rectal biopsy findings, as the surgical anastomosis would likely be near the prostate fields.

Multiple treatment paradigms were considered for the rectal cancer, including a long course of upfront chemoradiation to the rectum and prostate, followed by chemotherapy, or induction chemotherapy, followed by a short course of radiation to the rectum and prostate. Given the potential delay in treating the prostate cancer with the induction chemotherapy approach and the higher acute toxicity of a short radiation course, the decision was made to begin management with total neoadjuvant therapy (TNT).

**Figure 3:**
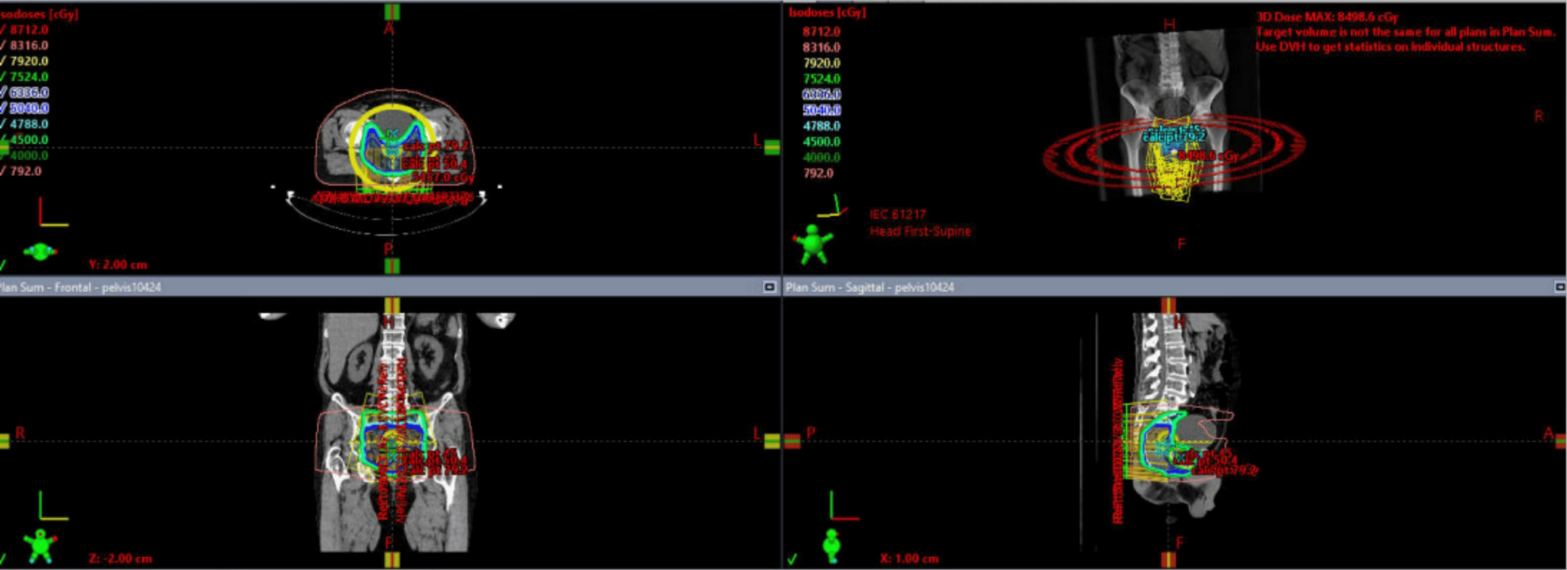
External beam radiation fields demonstrating estimated doses at each target volume.

The patient was prescribed three sequential radiation treatments (Figure 3). First, the patient received volumetric modulated arc therapy (VMAT) to the rectum, prostate, and lymph nodes at a dose of 4500 cGy given in 25 fractions of 180 cGy. This was followed by VMAT to the rectum and prostate at a dose of 540 cGy given in three fractions of 180 cGy. Finally, the patient received VMAT to the prostate at a dose of 2880 cGy given in 16 fractions of 180 cGy. Concurrent capecitabine 1500 mg was given throughout these treatment intervals.

Following the completion of chemoradiation, the patient received folinic acid, fluorouracil, and oxaliplatin (FOLFOX). No symptoms of acute toxicity, including diarrhea, melena, and rectal pain, were endorsed by the patient during treatment.

Outcomes

At the six-month follow-up, the patient reported mild intermittent constipation and bowel urgency but denied rectal pain or blood per rectum. Physical examination demonstrated the complete regression of the rectal tumor intraluminally. However, the MRI of the pelvis revealed residual tumor with a small T2 signal focus of restricted diffusion. This finding was thought to represent partial treatment response, requiring possible curative surgical intervention. The patient’s repeat PSA measured <0.1 ng/mL, and he denied hematuria and dysuria; accordingly, active surveillance with PSA every three months and serial MRI was opted for. The MRI of the pelvis with and without contrast, performed five months later, demonstrated a complete response.

## Discussion

The concurrent incidence of prostate and rectal cancer in this patient highlights important aspects of the treatment of primary malignancies in close proximity. Consistent with other cancer types, rectal and prostate cancer are managed based on tumor stage and metastatic potential. While endoscopic removal is adequate for low-grade malignant rectal polyps with negative margins and no lymphovascular involvement, those demonstrating advanced clinical and histological features require more extensive treatment. Long-course neoadjuvant chemoradiation is recommended for patients with T3, N1 disease [10]. These regimens typically include fluoropyrimidine-based chemotherapy and pelvic radiation that aim to downstage the tumor, improve local control, and increase the likelihood of organ preservation. For patients who achieve a complete clinical response (cCR), commonly defined as the absence of a mass, small mucosal irregularities (e.g., ≤2 cm in diameter) at endoscopy, and no metastatic nodes on MRI, after neoadjuvant therapy, a “watch-and-wait” strategy may be considered [10]. This approach involves close surveillance with regular digital rectal examinations, endoscopy, and MRI to monitor for any signs of recurrence.

Asare et al. reported that 64.4% of patients achieved cCR with TNT, and surgery-free survival rates were 77.3% at one year and 66.2% at two years [11]. Further supporting this strategy, the Organ Preservation in Patients With Rectal Adenocarcinoma Treated With Total Neoadjuvant Therapy (OPRA) trial demonstrated that TNT, followed by selective watch-and-wait, achieved organ preservation in 40%-54% of patients with stage II/III rectal adenocarcinoma, completely avoiding both surgery and the need for additional pelvic interventions that could complicate subsequent prostate cancer-directed therapy [12]. In addition, the Chemotherapy Alone or Chemotherapy Plus Radiation Therapy in Treating Patients With Locally Advanced Rectal Cancer Undergoing Surgery (PROSPECT) trial demonstrated that neoadjuvant FOLFOX chemotherapy with the selective use of chemoradiotherapy is non-inferior to standard chemoradiotherapy for locally advanced rectal cancer [13]. This has important implications for patients with synchronous pelvic malignancies, as 89.6% of patients in the FOLFOX group avoided pelvic radiation entirely, receiving chemotherapy alone prior to surgery. Only 9% required preoperative chemoradiotherapy due to inadequate tumor regression (<20%). Five-year disease-free survival was similar between groups (80.8% in FOLFOX versus 78.6% in chemoradiotherapy), and local recurrence rates were exceptionally low (1.6% versus 1.8%). Within this paradigm, an alternative strategy for our patient would have been to prioritize rectal cancer management with TNT or a PROSPECT-like neoadjuvant chemotherapy-first approach, deferring definitive prostate treatment until after the completion of rectal cancer therapy.

Prostatectomy or external beam radiation therapy (EBRT) is the standard curative treatment for all stage II prostate cancers [14]. Long-term follow-up studies have demonstrated significantly reduced prostate cancer-specific mortality and risk of metastases compared to watchful waiting [15]. However, it is associated with higher rates of urinary incontinence and erectile dysfunction [15]. EBRT provides similar prostate cancer-specific survival rates to radical prostatectomy, though with a slightly different side effect profile, including bowel and bladder dysfunction [16]. Additionally, for patients with unfavorable intermediate- or high-risk disease, EBRT is typically combined with ADT, with recommended durations ranging from 4-6 months for unfavorable intermediate-risk disease to 18-36 months for high-risk or locally advanced disease. The prolonged course of ADT may contribute to metabolic, cardiovascular, and quality-of-life effects, including fatigue, sarcopenia, and sexual dysfunction. In the setting of synchronous malignancy, the anticipated duration of hormone therapy becomes an important factor in shared decision-making. A prolonged course of ADT may overlap with rectal cancer-directed therapy and recovery, potentially compounding treatment-related toxicity and functional decline. In our case, the patient strongly preferred organ preservation, limiting the duration of systemic therapy and avoiding a prolonged course of ADT, so a short course of four months of ADT was selected to complement radiotherapy while minimizing overlapping toxicity and treatment duration.

When encountering a synchronous disease, a more integrated approach to treatment is necessary to ensure adequate treatment of both cancers while minimizing the length of intervention and adverse events. Pitfalls for the management of cancers located in close proximity include differing malignant timelines. Locally advanced stage IIIB rectal cancers have a reported five-year survival rate of 30%-60% [8], whereas 98.6% five-year survival has been observed in regional prostate cancer among men in the United States [17]. As such, targeting rectal cancer early has important implications for overall survival. One potential strategy would be to complete rectal cancer treatment first, followed by the delayed definitive management of the prostate. However, proceeding with the isolated neoadjuvant treatment of the rectum and prostate in series may allow for prostate cancer progression and iatrogenic complications following resection. Increased morbidity due to fibrosis and scarring has been reported from salvage prostatectomy after external beam radiation therapy (EBRT) compared to primary prostatectomy [8].

Moreover, in this patient, baseline comorbidities, including significant smoking and alcohol histories, made him a suboptimal candidate for prostatectomy, and prostatectomy after low anterior resection (LAR) would carry even higher risk of complications and functional decline. Combined rectal and prostate surgery would be substantially more extensive and complex, with higher perioperative risk and impact on the quality of life. Furthermore, prostate cancer requires a higher radiation dose for adequate disease-free survival; late toxicity related to such prostate boosts may result in anastomotic leak, fistula, or stricture following low anterior resection [7,8]. However, several studies have highlighted increased morbidity associated with surgical management alone. Doussot et al. reported that among patients undergoing curative-intent rectal cancer resection combined with prostate cancer management, the overall morbidity rate was 64%, with 20% experiencing severe surgical morbidity, including two deaths within 90 days of surgery after pelvic exenteration [9]. In addition to perioperative risk, definitive chemoradiation strategies offer the potential for organ preservation. For patients who would otherwise require abdominoperineal resection with the removal of the anal sphincter and permanent colostomy, radiation-based approaches may allow the preservation of anorectal anatomy and the avoidance of a permanent stoma. Meanwhile, the overall incidence of severe urinary or rectosigmoid sequelae from EBRT is approximately 3%, with severe anorectal injury requiring colostomy being less than 1% [18].

As such, the expansion of the target volume to treat multiple primary malignancies has demonstrated favorable outcomes in select cases. Ng et al. describe the use of definitive VMAT in a patient with synchronous cT2b N0 M0 prostate adenocarcinoma and cT3 N1 M0 rectal adenocarcinoma without subsequent radical prostatectomy or rectal resection [19]. Normal PSA and CEA levels, satisfactory bladder and bowel function, and no evidence of disease recurrence were found at one-year follow-up [19]. Notably, the patient received 50.4 Gy in 28 fractions to the pelvis and a boost of 24 Gy in 12 fractions to the prostate. Avoiding surgery after definitive pelvic radiation is notable, as operative intervention in this setting carries a high risk of morbidity due to radiation-induced fibrosis and impaired healing. Similarly, Siu et al. found no evidence of rectal tumor recurrence and significant reductions in prostate-specific antigen levels at one and two years post-therapy in two cases of rectal and prostate cancer treated with three-dimensional conformal radiotherapy simultaneously [20].

Importantly, in a retrospective study of synchronous rectosigmoid and prostate cancer, Jacobs et al. found that two of 19 patients had experienced late grade 3 genitourinary (GU) toxicity, and four of 19 patients had late grade 3 gastrointestinal (GI) toxicity when treated with median doses of 66 Gy and 50.4 Gy to the prostate and rectosigmoid, respectively [7]. These rates are higher than the severe late toxicity typically reported with prostate-only radiotherapy at similar doses, where grade ≥3 GU and GI events generally occur in fewer than 5% of the patients, and they lie at or slightly above the upper range of severe late GI toxicity (up to ~19%) described after standard rectal chemoradiation [21,22]. Thus, regional toxicity is necessary to consider with the expansion of target volumes. In contrast to the higher rates of late toxicity reported in previous studies treated with concomitant prostate and rectosigmoid radiotherapy, our patient has, to date, maintained good urinary and bowel function without severe late toxicity while achieving the local control of both primaries with a lower pelvic dose, limited-duration ADT, and an organ‑preserving strategy. Long-term follow-up and monitoring will be necessary for determining the efficacy and safety of the dose given to the present patient.

## Conclusions

We describe a patient with synchronous stage IIIB, cT3, cN1, cM0 rectal adenocarcinoma and stage IIC, cT1, cN0, cM0 prostate adenocarcinoma. The diagnostic workup demonstrated histologically distinct cancers; however, the etiological relationship between the two malignancies cannot be ascertained. The patient was treated with comprehensive VMAT with capecitabine to the rectum, prostate, and regional lymph nodes, consistent with radiation plans described in previous reports, along with ADT for the prostate cancer and FOLFOX for the rectal cancer. Accordingly, the expansion of target volumes may adequately treat cancers in close proximity to each other. This method demonstrated limited toxicity, suggesting a favorable safety profile. Continuous investigations on the safety and comparative effectiveness of such treatments are needed to establish standard management strategies for synchronous prostate and rectal cancer.
